# Biomechanical and functional performance differences in individuals with and without pes planus: A systematic review and modified GRADE evidence synthesis protocol

**DOI:** 10.1016/j.mex.2026.104101

**Published:** 2026-08-16

**Authors:** Fatimah W. Abdelrahman, Kalyana Chakravarthy Bairapareddy, Ashokan Arumugam

**Affiliations:** a Department of Physiotherapy, College of Health Sciences, University of Sharjah, Sharjah, 27272, United Arab Emirates; b Neuromusculoskeletal Rehabilitation Research Group, RIMHS–Research Institute of Medical and Health Sciences, University of Sharjah, Sharjah, 27272, United Arab Emirates; c Sustainable Engineering Asset Management Research Group, RISE—Research Institute of Sciences and Engineering, University of Sharjah, Sharjah, P.O. Box 27272, United Arab Emirates; d Department of Physiotherapy, Manipal College of Health Professions, Manipal Academy of Higher Education, Manipal, India

**Keywords:** Flatfoot, Pes planus, Foot biomechanics, Gait analysis, Functional performance, Plantar pressure

## Abstract

**Background:**

Pes planus (flat foot) is characterized by a reduction of the medial longitudinal arch and alterations in foot biomechanics that may influence gait, balance, and functional performance. However, existing evidence regarding its biomechanical and functional consequences remains inconsistent. This systematic review aims to examine differences in biomechanics and functional performance among individuals with pes planus and those with normal arches.

**Methods:**

This review will include individuals of any age, sex, diagnosed with pes planus using validated criteria, compared with individuals with normal foot arches. Observational and experimental studies reporting biomechanical or functional outcomes will be included. Primary outcomes will comprise biomechanical measures, including kinematic, kinetic, spatiotemporal, plantar pressure, and muscle activity measures. Secondary outcomes will include, but not be limited to, functional performance measures such as balance and agility. A systematic search will be conducted across PubMed, Scopus, Web of Science, SPORTDiscus, EMBASE, and CINAHL, supplemented by grey literature searches in ProQuest, OpenGrey, and Google Scholar. Two independent reviewers will perform study selection, data extraction, and quality assessment using the Downs and Black index. Effect sizes will be reported as standardized mean differences or odds ratios with 95% confidence intervals, and the certainty of evidence will be evaluated using a modified GRADE approach.

**Systematic review registration number:**

CRD420261331845.

## Graphical abstract

**Figure:**
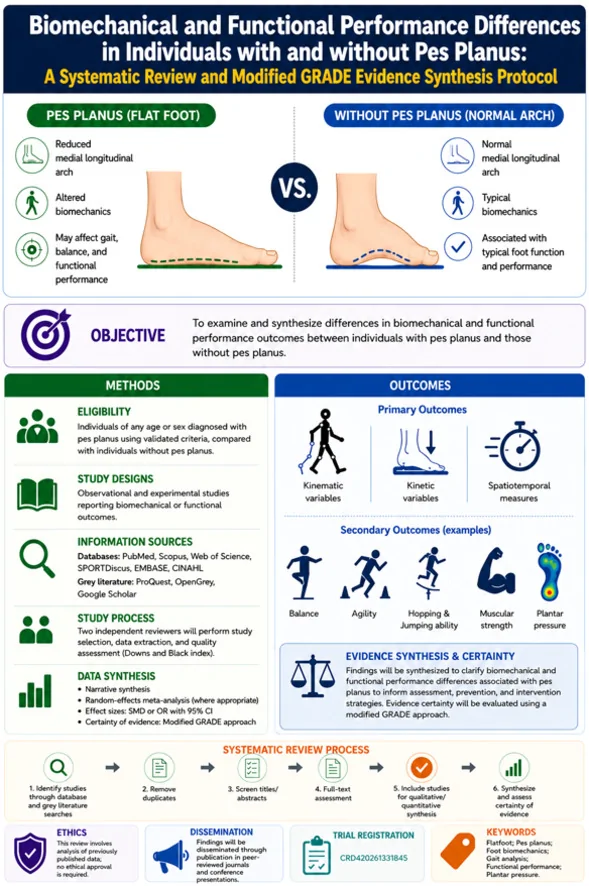

## Background

Pes planus, is defined by a reduction of the medial longitudinal arch, often accompanied by excessive pronation and altered alignment of the foot segments [1]. It is a common condition, affecting approximately 25% of the general population [2,3]. This structural alteration is clinically important because the medial longitudinal arch contributes to shock absorption, load transfer, and efficient gait mechanics [4].

Variations in the medial longitudinal arch (MLA) height, specifically quantified using the Navicular Drop Test with a threshold greater than 10 mm [5,6], could impact lower-limb biomechanics and functional capacity in adults [7]. Moreover, during gait, multi-segment foot analysis using the Oxford Foot Model has demonstrated that individuals with pes planus exhibit increased rearfoot eversion, altered forefoot motion, and differences in midfoot kinematics compared with individuals with normal arches [8]. In addition, foot posture has been shown to influence dynamic gait patterns, with planus feet displaying altered joint kinematics across the stance phase when compared with normal and cavus foot types [4,9]. These deviations may contribute to inefficient load distribution and compensatory movement strategies throughout the lower kinetic chain [9,10].

From a functional perspective, these biomechanical alterations may translate into observable differences in performance [11]. Comparative studies have shown that individuals with pes planus demonstrate differences in alignment, stability, and force generation when compared with individuals with normal arches [[12], [13], [14]]. Impairments in balance and neuromuscular control have also been associated with pes planus [7,15]. Reduced dynamic postural stability may be linked to decreased activation or efficiency of intrinsic foot muscles and altered proprioceptive input [15]. Evidence from intervention studies suggests that strengthening of intrinsic foot muscles can improve foot function and balance, highlighting the functional importance of these structures [16,17]. Furthermore, recent biomechanical studies suggest that gait-retraining strategies, particularly modification of the foot progression angle, may help modify lower-limb movement patterns and loading in individuals with flexible pes planus [18]. As alterations in foot progression angle have been associated with changes in ankle and knee kinematics, kinetics, and internal knee loading [19]. These findings support the potential use of individualized gait modification alongside exercise-based rehabilitation, while further clinical research is needed to better understand its practical benefits.

Although not all individuals with pes planus are symptomatic, some may experience pain, reduced functional performance, and an increased risk of overuse injuries, particularly with higher physical demands [20]. Previous literature has predominantly focused on intervention-based outcomes rather than directly comparing baseline differences between individuals with and without pes planus [[21], [22], [23]]. Consequently, there remains a need to systematically review and synthesize existing evidence to better understand how pes planus influences both biomechanical and functional outcomes [[24], [25], [26], [27]]. The primary aim of this systematic review is to evaluate and synthesize evidence on differences in lower-limb biomechanics during gait between individuals with pes planus and those with normal medial longitudinal arches. The secondary aim is to evaluate differences in functional performance between individuals with pes planus and those with normal medial longitudinal arches. The findings of this review could have important implications for clinical practice in the management of individuals with pes planus. Incorporating evidence on biomechanical and functional alterations into assessment and intervention strategies may support personalized rehabilitation, guide clinical decision-making, and help prevent secondary complications associated with abnormal foot mechanics.

## Description of protocol

### Study design

This protocol of the systematic review adheres to the recommendation of the preferred reporting items for systematic reviews and meta-analysis protocols (PRISMA-P 2015) guidelines (**supplementary file 1: PRISMA-P 2015 Checklist**) [28]. The protocol has been registered a priori in the PROSPERO database, the International prospective register of systematic reviews (CRD420261331845).

### Eligibility criteria

The eligibility criteria are based on the PECOS elements of our questions described in (Table 1)*.* This review will include studies comparing individuals with pes planus and those with normal foot arches [6,29]. Both observational and experimental studies reporting relevant biomechanical or functional outcomes will be considered [[30], [31], [32]]. Studies including participants with other conditions affecting foot function (e.g., neurological or systemic diseases), and non–peer-reviewed publications will be excluded. The search will be limited to English-language publications because of practical limitations in the availability of translation resources and the need to ensure accurate assessment of methodological details and study findings. However, this restriction may introduce language bias and potentially result in the omission of relevant evidence published in other languages. This limitation will be acknowledged when interpreting the findings of the review.

**Table 1:** Eligibility Criteria.

PECOS	Inclusion Criteria	Exclusion Criteria	
**Population**	Individuals with pes planus (Navicular Drop >10 mm or FPI-6 > 6) and individuals with normal arches (Navicular Drop <10 mm, FPI-6 ≤ 6).		Individuals with other foot deformities, history of foot/ankle surgery, acute lower-limb injuries, or neurological/systemic conditions affecting foot function (e.g., cerebral palsy, peripheral neuropathy).
**Exposure**	Presence of pes planus.		Absence of pes planus.
**Comparator**	Individuals with normal foot arches.		N/A
**Outcomes**	**Primary:** Lower-limb biomechanics: (lower limb kinematics, Kinetics, and spatiotemporal parameters, plantar pressure, and muscle activity measures). **Secondary:** Functional performance: (balance, agility, hopping/jumping, and muscular strength).		Outcomes unrelated to foot biomechanics or function, or from interventions rather than baseline measures.
**Study Design**	Observational and experimental studies reporting baseline outcomes.		Case reports, case series, reviews, editorials, conference abstracts without full data, non-peer-reviewed publications; non-English language studies.

### Outcome measures

The primary outcomes will comprise lower-limb biomechanical measures, as these directly reflect the biomechanical alterations associated with pes planus and address the primary objective of this review. These outcomes will be categorized into kinematic, kinetic, spatiotemporal, plantar pressure, and muscle activity domains. Kinematic outcomes will include measures such as peak ankle eversion angle, expressed in degrees; spatiotemporal outcomes will include gait velocity, expressed in meters per second; kinetic outcomes will include peak vertical ground reaction force, normalized to body weight; Plantar pressure outcomes will include peak plantar pressure and pressure-time integral. Muscle activity outcomes will include lower-limb muscle activation measured using surface electromyography (EMG), including activation amplitude (%MVC) where reported. Secondary outcomes will comprise functional performance measures, including balance, expressed as normalized reach distance; agility, expressed as completion time in seconds; hopping performance, expressed as hop distance in meters; jumping performance, expressed as jump height in centimeters; and muscular strength, expressed as force normalized to body weight.

### Information sources

A systematic search will be conducted across PubMed, Scopus, Web of Science, SPORTDiscus, EMBASE, and CINAHL, supplemented by grey literature searches in ProQuest for dissertations and theses, OpenGrey, and Google Scholar. To maintain feasibility and relevance, the Google Scholar search will be limited to the first 200 results sorted by relevance. Databases will be searched for articles published from 4 January 2016 and up to April 2026. This timeframe was selected to provide a more current representation of lower-limb biomechanics and functional performance in individuals with pes planus, while maintaining a sufficiently broad period for identifying relevant evidence. Studies published before 2016 were therefore excluded to maintain a contemporary focus of the review.

### Search strategy

A systematic search will be conducted across major electronic databases, supplemented by grey literature source presented in (Table 2). Keywords were selected based on PECO frameworks. The PubMed search strategy is presented in (Table 3), and the full search strings for all databases are provided in (**supplementary file 2**). The search approach incorporates Boolean operators, including OR AND, as well as truncation and wildcard symbols, to ensure a comprehensive and sensitive retrieval of relevant studies.

**Table 2:** PECO-Based Search Strategy Keywords.

PECO	Concept	Keywords / Search Terms
**P**	Population (Foot Structure)	1. flat foot 2. pes planus 3. low arch 4. Flat Foot* 5. 'flat foot'/exp* 6. (MH Flat Foot+)* 7. OR 2 OR 3 OR 4 OR 5 OR 6
**E**	Exposure (Pes Planus Presence)	8. pes planus 9. flat foot 10. low arch 11. arch collapse 12. pronated foot 13. 8 OR 9 OR 10 OR 11 OR 12
**C**	Comparator (Normal Arch)	14. normal arch 15. neutral foot 16. normal foot posture 17. arch height 18. navicular drop 19. Foot Posture Index OR FPI-6 20. OR 15 OR 16 OR 17 OR 18 OR 19
**O (Primary)**	Biomechanics / Gait	21. biomechanic 22. biomechanics* 23. 'biomechanics'/exp* 24. (MH Biomechanical Phenomena+)* 25. joint kinematic* 26. kinematic* 27. kinetic* 28. hip 29. knee 30. ankle 31. spatiotemporal gait parameter* 32. gait OR walking OR locomotion 33. ground reaction force* 34. plantar pressure 35. EMG OR electromyograph* 36. Electromyography* 37. OR/21–36
**O (Secondary)**	Functional Performance	38. functional performance 39. 'functional performance'/exp* 40. (MH Physical Performance+)* 41. balance 42. agility 43. hop test 44. jump test 45. muscular strength 46. strength 47. OR/38–46

(*) indicates controlled vocabulary terms (MeSH in PubMed, Emtree in Embase, CINAHL Headings).

**Table 3:** Search strategy used in PubMed (inception to 2026). Search date: 9 April 2026.

Number	Search items
**1**	"Flat Foot"[Mesh] OR pes planus OR "low arch"
**2**	"Biomechanics"[Mesh] OR "joint kinematics" OR hip OR knee OR ankle OR "spatiotemporal gait parameters" OR "ground reaction forces" OR "plantar pressure" OR EMG OR electromyography OR "functional performance" OR balance OR agility OR "hop test" OR "jump test" OR "muscular strength" OR strength
**3**	1 AND 2

[Mesh] indicate a Medical Subject Headings (MeSH) term.

### Study records

References will be organized using Mendeley reference management software [33] and subsequently imported into the free version of Rayyan, a collaborative platform designed to facilitate systematic and literature reviews [34]. Duplicate records will be automatically removed within Rayyan. Two reviewers (FW, KCB) will independently screen the titles and abstracts of all retrieved records. Articles that clearly do not meet the eligibility criteria will be excluded, while those that may potentially be relevant will be classified as “maybe.” Each reviewer will then independently assess the full set of “maybe” articles to confirm their potential eligibility. Following this stage, blinding between reviewers will be removed, and a consensus meeting will be held to resolve any discrepancies in the pre-selection list. If agreement cannot be reached, a third reviewer (AA) with experience in systematic reviews will be consulted.

The two reviewers (FW, KCB) will then independently examine the full texts of all eligible articles to determine final inclusion based on the predefined criteria. Disagreements at this stage will also be resolved through discussion or consultation with a third reviewer if necessary. For studies meeting the inclusion criteria, two reviewers will extract the relevant data, including participant demographics, methods of pes planus assessment, biomechanical and functional performance outcomes, and reported statistical results. A PRISMA 2020 flow diagram will be used to report the selection process [28]. Disagreements at this stage will also be resolved through discussion or consultation with a third reviewer if necessary.

### Data items

Data corresponding to all PECOS items will be extracted from each included study (Table 1). This will include participant characteristics such as age, sex, and severity (grades) of pes planus. Biomechanical outcomes related to kinetic, kinematics, muscle activity, spatiotemporal parameters, and functional performance measures will be collected. Additionally, study-level information including study design, objectives, sample size, and year of publication will be recorded to support synthesis and comparison across studies.

### Risk of bias assessment

Risk of bias will be assessed using the Downs and Black index. The Downs and Black checklist will be used to evaluate the methodological quality of both randomized and non-randomized comparative studies [35]. This instrument consists of 28 items assessing reporting, external validity, internal validity including bias and confounding, and statistical power, where higher scores reflect higher study quality [35,36]. For observational studies, eight items (Questions 4, 8, 14, 15, 19, 23, 24, and 27) are excluded, while an additional two items (Questions 9 and 26) are excluded for cross-sectional studies. Consequently, studies with a longitudinal design can achieve a maximum score of 20, whereas cross-sectional studies can achieve up to 18 points [37].The checklist has demonstrated acceptable measurement properties, with evidence supporting its reliability and validity for assessing both observational studies and experimental studies [36,38]. In the present review, studies will be classified as low quality if they meet <60% of applicable criteria, moderate quality if they meet 60–74%, and high quality if they meet 75% or more [37]. Two reviewers will independently complete the assessment, with discrepancies resolved through discussion, and a third reviewer will verify the final scores to ensure accuracy.

### Data synthesis

A narrative summary of population characteristics, biomechanical and functional performance outcomes, and risk of bias ratings using the Downs and Black index of the individual studies will be presented in figures and tabular format as required [35]. Observational and experimental studies will be synthesized separately to account for differences in study design, methodological characteristics, and risk of bias. Quantitative synthesis will be conducted within each study design when sufficient clinical, methodological, and statistical homogeneity exists among studies reporting the same or sufficiently comparable outcomes. Where data or methodological details are missing or unclear, the corresponding authors of the included studies will be contacted for clarification where feasible. If no response is received or the information remains unavailable, analyses will be conducted using the available data, and the potential impact of missing information on the findings will be acknowledged in the review. When multiple measurements of the same outcome are reported within a study, including different instruments, testing protocols, or time points, all relevant data will initially be extracted. For quantitative synthesis, the measurement that most closely corresponds to the predefined outcome and is most comparable across studies will be prioritized. Where measurements cannot be appropriately prioritized, the findings will be considered in the narrative synthesis.

A meta analysis using a random effects model will be conducted when pooling is supported by the comparability of study populations, study designs, interventions or exposures, outcome measures, and effect estimates [39,40]. The decision to conduct a meta analysis will therefore be based on the characteristics and homogeneity of the available evidence [41,42]. Similar to previous reports [40,41], pooled estimates of effect sizes will be calculated using appropriate statistical measures, including standardized mean differences (SMD) for continuous outcomes, such as kinematics, spatiotemporal, kinetics, and functional performance measures, and odds ratios for categorical outcomes, with 95% confidence intervals (CIs) [42]. These pooled estimates will be presented in forest plots [43]. Statistical heterogeneity will be assessed using the I² statistic, with I² > 40% considered indicative of substantial heterogeneity [42], and τ² will be used to account for between study variability [44].Subgroup meta analyses will be conducted where sufficient data are available to support meaningful comparisons, according to predefined participant characteristics, including age (<18 years vs. ≥18 years), sex, and pes planus severity (grades). Age will be examined to account for potential differences across age groups, sex to explore potential differences between male and female participants, and pes planus severity to assess whether the magnitude of biomechanical and functional alterations varies with severity. Flexible versus rigid pes planus and symptomatic versus asymptomatic presentations will be explored through subgroup or narrative analyses when reported, as these characteristics may also contribute to variation in findings. Other potential sources of heterogeneity, including assessment method, testing condition (barefoot or shod), task type (walking or running), gait speed, and foot model configuration, will be considered during data synthesis and interpretation.

Sensitivity analyses will be performed by excluding studies at high risk of bias to evaluate the robustness of findings. Where at least 10 studies are available [45], publication bias and potential small study effects will be assessed using funnel plots. Where funnel plot asymmetry is observed, potential explanations for the asymmetry will be explored, and trim and fill analyses will be applied where appropriate to assess the potential impact of missing studies [46]. Where the available evidence does not support a meta analysis, each outcome will be synthesized narratively to clarify the clinical relevance of pes planus related biomechanical and functional alterations and to identify gaps in the current literature.

### Confidence in synthesized evidence

The confidence in the body of evidence will be evaluated using a modified GRADE (Grading of Recommendations, Assessment, Development, and Evaluation) approach, which provides a transparent framework for assessing and summarizing evidence [47,48]. This approach allows a systematic appraisal of each outcome, considering factors such as study design, risk of bias, consistency of results, precision, directness of evidence, and other factors such as publication bias [49]. Evidence will be rated across four levels: very low, low, moderate, and high to reflect the overall certainty of the findings and inform interpretation of the biomechanical and functional outcomes related to pes planus [50].

As the review is expected to include predominantly observational studies, the initial certainty of evidence will be rated as low [50]. Evidence may be upgraded by one or two levels when predefined criteria are met, including a large or very large magnitude of effect, a clear dose–response gradient, or plausible residual confounding that would be expected to reduce the observed effect [48,50]. Certainty of the evidence will be assessed separately for each outcome, with consideration of differences in measurement methods, testing protocols, and operational definitions across biomechanical and functional performance outcomes. Two reviewers will independently assess the certainty of evidence, with disagreements resolved through discussion and, where necessary, consultation with a third reviewer. The modified approach will retain the standard GRADE four-level certainty scale while adapting the decision-making process to the characteristics of observational biomechanical and functional performance evidence, particularly variations in outcome measurement and clinical heterogeneity across studies [47,48].

## Protocol validation

This systematic review protocol was developed and reported in accordance with the PRISMA-P (Preferred Reporting Items for Systematic Review and Meta-Analysis Protocols) guidelines. The completed PRISMA-P checklist is provided as Supplementary File 1 to support protocol validation and demonstrate adherence to established reporting standards. This protocol is currently at Version 2 and represents the stable version following revisions made during the development and review process. Following PROSPERO registration (CRD420261331845), any important amendment to the protocol will be documented by recording the date of the amendment, the specific section affected, a description of the change, and the rationale for making the change. Where applicable, the PROSPERO record will be updated to reflect substantive changes to the review methods. All-important amendments will also be reported in the final systematic review, including a clear description of the changes and their potential impact on the review. Minor editorial or administrative changes that do not affect the methodology, eligibility criteria, analysis plan or interpretation of the review will be documented but will not require a formal amendment.

## Limitations

Potential limitations of this review include clinical and methodological heterogeneity arising from differences in participant characteristics, pes planus classification and severity, diagnostic criteria, and outcome assessment methods. Such variability may limit the comparability of studies and influence the interpretation of the findings. In addition, restricting the review to English-language publications may result in the exclusion of relevant studies, and the possibility of publication bias cannot be ruled out.

## CRediT authorship contribution statement

**Fatimah W. Abdelrahman:** Conceptualization, Data curation, Formal analysis, Investigation, Methodology, Project administration, Writing – original draft, Writing – review & editing. **Kalyana Chakravarthy Bairapareddy:** Data curation, Formal analysis, Investigation, Methodology, Supervision, Writing – review & editing. **Ashokan Arumugam:** Formal analysis, Methodology, Supervision, Validation, Writing – review & editing.

## Declaration of interests

The authors declare that they have no known competing financial interests or personal relationships that could have appeared to influence the work reported in this paper.

## Data Availability

No data was used for the research described in the article.

## Appendix Supplementary materials

Image, application 1

Image, application 2
