# Supplementary material for: Biomechanical and functional performance differences in individuals with and without pes planus: A systematic review and modified GRADE evidence synthesis protocol
Source: MethodsX. 2026 Aug 16;17:104101. doi: 10.1016/j.mex.2026.104101 (PMC13524489; doi:10.1016/j.mex.2026.104101)
Supplement: Supplementary file 2 [file mmc2.docx]

**Supplementary Material 2. Database-Specific Search Strategies**

| **Database** | **Search Strategy** |
| --- | --- |
| PubMed | ("Flat Foot"[Mesh] OR pes planus OR "low arch") AND ("Biomechanics"[Mesh] OR "joint kinematics" OR hip OR knee OR ankle OR "spatiotemporal gait parameters" OR "ground reaction forces" OR "plantar pressure" OR EMG OR electromyography OR "functional performance" OR balance OR agility OR "hop test" OR "jump test" OR "muscular strength" OR strength) |
| Scopus (Title/Abstract/Keywords) | TITLE-ABS-KEY ("flat foot" OR "pes planus" OR "low arch") AND TITLE-ABS-KEY("biomechanics" OR "joint kinematics" OR hip OR knee OR ankle OR "spatiotemporal gait parameters" OR "ground reaction forces" OR "plantar pressure" OR EMG OR electromyography OR "functional performance" OR balance OR agility OR "hop test" OR "jump test" OR "muscular strength" OR strength) |
| Web of Science (TS) | TS=("flat foot" OR "pes planus" OR "low arch") AND TS=("biomechanics" OR "joint kinematics" OR hip OR knee OR ankle OR "spatiotemporal gait parameters" OR "ground reaction forces" OR "plantar pressure" OR EMG OR electromyography OR "functional performance" OR balance OR agility OR "hop test" OR "jump test" OR "muscular strength" OR strength) |
| SPORTDiscus | ("flat foot" OR "pes planus" OR "low arch") AND (biomechanic* OR "joint kinematic*" OR hip OR knee OR ankle OR "spatiotemporal gait parameter*" OR "ground reaction force*" OR "plantar pressure" OR EMG OR electromyograph* OR "functional performance" OR balance OR agility OR "hop test" OR "jump test" OR "muscular strength" OR strength) |
| Embase (Emtree + Free Text) | ('flat foot'/exp OR 'pes planus' OR 'low arch') AND ('biomechanics'/exp OR joint kinematic* OR hip OR knee OR ankle OR spatiotemporal gait parameter* OR ground reaction force* OR plantar pressure OR emg OR electromyograph* OR 'functional performance'/exp OR balance OR agility OR 'hop test' OR 'jump test' OR muscular strength OR strength) |
| CINAHL (Headings + TI/AB) | (MH "Flat Foot+" OR TI "flat foot" OR AB "flat foot" OR TI "pes planus" OR AB "pes planus" OR TI "low arch" OR AB "low arch") AND (MH "Biomechanical Phenomena+" OR MH "Physical Performance+" OR TI biomechanic* OR AB biomechanic* OR TI "joint kinematic*" OR AB "joint kinematic*" OR TI hip OR AB hip OR TI knee OR AB knee OR TI ankle OR AB ankle OR TI "spatiotemporal gait parameter*" OR AB "spatiotemporal gait parameter*" OR TI "ground reaction force*" OR AB "ground reaction force*" OR TI "plantar pressure" OR AB "plantar pressure" OR TI EMG OR AB EMG OR TI electromyograph* OR AB electromyograph* OR TI "functional performance" OR AB "functional performance" OR TI balance OR AB balance OR TI agility OR AB agility OR TI "hop test" OR AB "hop test" OR TI "jump test" OR AB "jump test" OR TI "muscular strength" OR AB "muscular strength" OR TI strength OR AB strength) |
| Grey Literature (Google Scholar, ProQuest, OpenGrey) | "flat foot" OR "pes planus" OR "low arch" AND biomechanics OR "joint kinematics" OR hip OR knee OR ankle OR gait OR "ground reaction forces" OR "plantar pressure" OR EMG OR electromyography OR "functional performance" OR balance OR agility OR "hop test" OR "jump test" OR "muscular strength" OR strength. First 200 results from Google Scholar will be screened, along with all relevant records retrieved from ProQuest and OpenGrey. |
